# Supplementary material for: Silicene nanomesh
Source: Sci Rep. 2015 Mar 13;5:9075. doi: 10.1038/srep09075 (PMC4649852; doi:10.1038/srep09075)
Supplement: Supplementary Information [file srep09075-s1.pdf]

Supplementary Information

Silicene Nanomesh

Feng Pan[1, 4, †], Yangyang Wang[3, †] Kaili Jiang[3, †], Zeyuan Ni[3], Jianhua Ma[5], Jiaxin Zheng[6], Ruge Quhe[3,7], Junjie Shi,[3] Jinbo Yang,[2,3] Changle Chen[1], and Jing Lu[2,3,*]

[1]Shaanxi Key Laboratory of Condensed Matter Structures and Properties, School of Science, Northwestern Polytechnical University, Xi'an 710072, P. R. China

[2]Collaborative Innovation Center of Quantum Matter, Beijing 100871, P. R. China

[3]State Key Laboratory for Mesoscopic Physics and Department of Physics, Peking University, Beijing 100871, P. R. China

[4]School of Physics and Telecommunication Engineering, Shaanxi University of Technology, Hanzhong 723001, P. R. China

[5]School of Physics and Nuclear Energy Engineering, Beihang University, Beijing 100191, P. R. China

[6]School of Advanced Materials, Peking University, Shenzhen Graduate School, Shenzhen 518055, P. R. China

[7]Academy for Advanced Interdisciplinary Studies, Peking University, Beijing 100871, P. R. China

[†]These authors contributed equally to this work.

[*]Address correspondence to jinglu@pku.edu.cn

Movie S1. Molecule dynamic process of the [R = 1, W = 2] SNM at 1000 K.

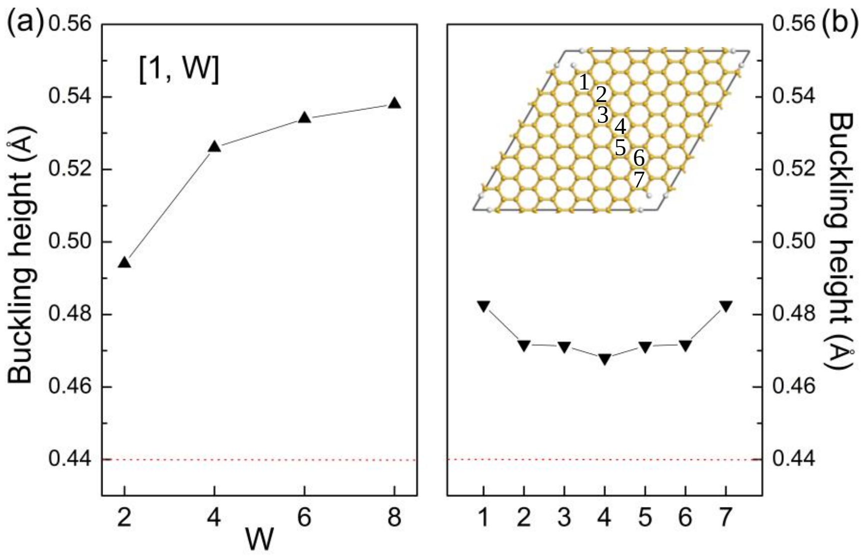

Figure S1. (a) Buckling height (Δ) of silicon atoms in the edge of holes as a function of W at R = 1. (b) Δ of different hexagonal Si atoms locations. The insert is the unit cell of [1, 8] silicene nanomesh. The red dashed line indicates the Δ of silicene.

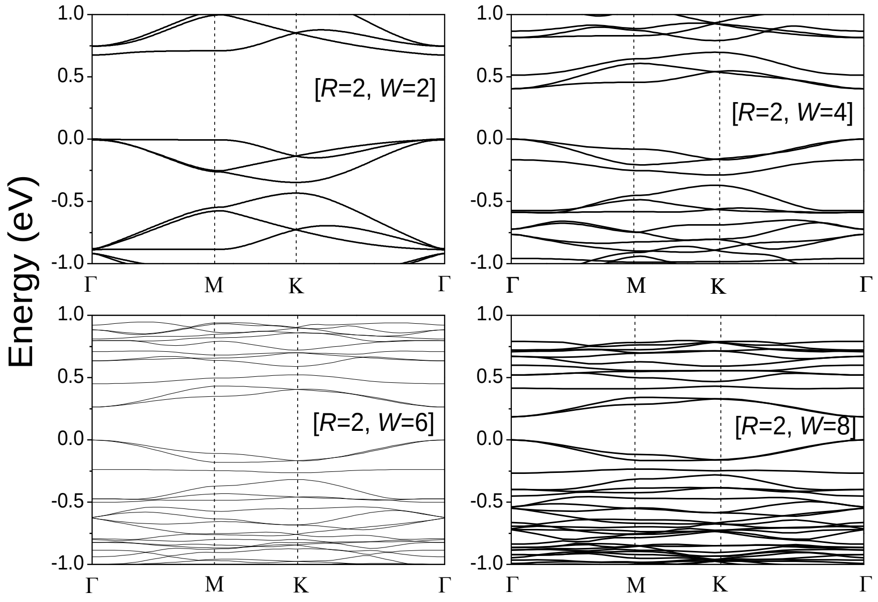

Figure S2. (a) Energy band structures of silicene nanomeshes with R = 2 and W = 2, 4, 6, and 8.

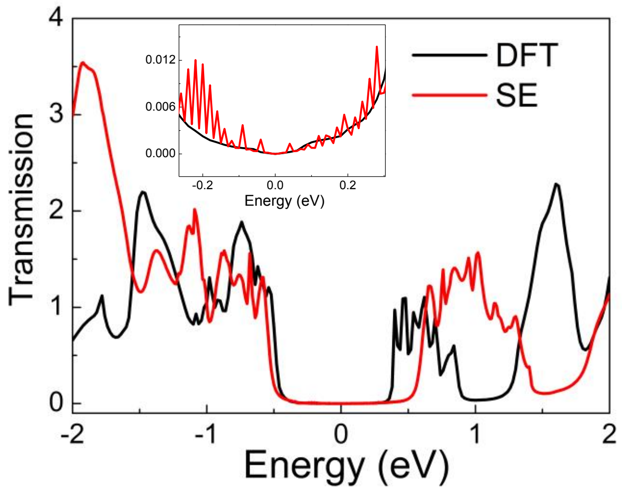

Figure S3. Benchmark of the transmission spectra of the silicene nanomesh transistor with L_{gate} = 6.5 nm calculated by the DFT and SE methods. Inset: zoom-in of the transmission spectra near E_f. The bias voltage and gate voltage are zero.

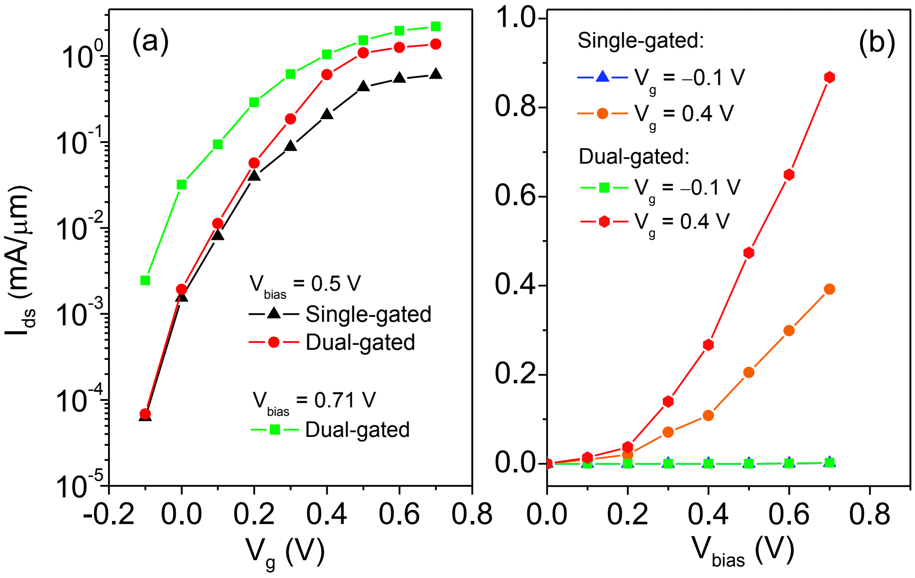

Figure S4. (a) Transfer characteristics of the 7.8 nm dual-gated SNM FETs at V_{bias} = 0.5 and 0.71 V compared with that of the single-gated SNM FETs with the same L_{gate} at V_{bias} = 0.5 V. (b) Output characteristics for the 7.8 nm single- and dual-gated SNM FETs at V_g = −0.1 and 0.4 V.
